# Electrocortical activity associated with subjective communication with the deceased

**DOI:** 10.3389/fpsyg.2013.00834

**Published:** 2013-11-20

**Authors:** Arnaud Delorme, Julie Beischel, Leena Michel, Mark Boccuzzi, Dean Radin, Paul J. Mills

**Affiliations:** ^1^Institute of Noetic SciencesPetaluma, CA, USA; ^2^Institute of Neural Computation, SCCN, University of CaliforniaSan Diego, La Jolla, CA, USA; ^3^Windbridge InstituteTucson, AZ, USA; ^4^Department of Psychiatry, University of CaliforniaSan Diego, La Jolla, CA, USA

**Keywords:** mediums, EEG, intuition, mental states, transcendence

## Abstract

During advanced meditative practices, unusual perceptions can arise including the sense of receiving information about unknown people who are deceased. As with meditation, this mental state of communication with the deceased involves calming mental chatter and becoming receptive to subtle feelings and sensations. Psychometric and brain electrophysiology data were collected from six individuals who had previously reported accurate information about deceased individuals under double-blind conditions. Each experimental participant performed two tasks with eyes closed. In the first task, the participant was given only the first name of a deceased person and asked 25 questions. After each question, the participant was asked to silently perceive information relevant to the question for 20 s and then respond verbally. Responses were transcribed and then scored for accuracy by individuals who knew the deceased persons. Of the four mediums whose accuracy could be evaluated, three scored significantly above chance (*p* < 0.03). The correlation between accuracy and brain activity during the 20 s of silent mediumship communication was significant in frontal theta for one participant (*p* < 0.01). In the second task, participants were asked to experience four mental states for 1 min each: (1) thinking about a known living person, (2) listening to a biography, (3) thinking about an imaginary person, and (4) interacting mentally with a known deceased person. Each mental state was repeated three times. Statistically significant differences at *p* < 0.01 after correction for multiple comparisons in electrocortical activity among the four conditions were obtained in all six participants, primarily in the gamma band (which might be due to muscular activity). These differences suggest that the impression of communicating with the deceased may be a distinct mental state distinct from ordinary thinking or imagination.

## Introduction

Individuals who report experiencing communication with deceased persons are traditionally called mediums. During a typical mediumship reading, a medium conveys messages from deceased persons to the living (i.e., sitters). There are two types of mediumship: mental and physical. In mental mediumship, communication with deceased persons is experienced “through *interior* vision or hearing, or through the spirits *taking over* and controlling their bodies or parts thereof, especially … the parts required for speech and writing” (Gauld, 1982, p. 4). During physical mediumship, the experienced communication “proceeds through paranormal physical events in the medium's vicinity” (Gauld, 1982, p. 4), which have included reports of independent voices, rapping sounds on walls or tables, and movement of objects (Fontana, 2005).

The practice of mediumship can be traced to primeval times, where shamans in the earliest communities provided guidance to the tribe through purported communication with the spirit world. Mediumship is generally not regarded in a favorable light by traditional Judeo-Christian-Islamic religions, although it is referred to as a genuine phenomenon in sacred texts (e.g., Samuel 28:1–25 in the Old Testament). Modern societies have seen a recent and dramatic increase in the portrayal of mental mediums in popular culture and a similar increase in practicing mediums offering their services.

The scientific study of mediumship began in the nineteenth century. Books like *The Mediums' Book* published by Kardec (1861/2009) popularized a rational approach to studying these phenomena. Members of the British and American Societies for Psychical Research, which studied mediumship in the heyday of spiritualism in the late 1800s, included psychologist William James, physicist Oliver Lodge, and physiologist and Nobel laureate Charles Richet. William James noted that the study of mediumship “cries aloud for serious investigation” (cited in Moreira-Almeida, 2012, p. 194) because although many mediums were found to be fraudulent, some who were studied under strictly controlled conditions appeared to have genuine access to information by non-ordinary means.

Scientific research on mediumship has also witnessed a small resurrection within the last decade. More recent research has examined the accuracy of statements provided by mediums under double- and triple-blind conditions (e.g., Roy and Robertson, 2004; O'Keefe and Wiseman, 2005; Beischel and Schwartz, 2007; Jensen and Cardeña, 2009; Kelly and Arcangel, 2011) as well as mediums' phenomenology (e.g., Rock and Beischel, 2008; Rock et al., 2009), psychology (e.g., Roxburgh and Roe, 2011), neurobiology (e.g., Hageman et al., 2010), and the therapeutic potential of mediumship readings for the bereaved (Beischel et al., in press). Recent research has also confirmed previous findings that mediumship is not associated with conventional dissociative experiences, pathology, dysfunction, psychosis, or over-active imaginations (Roxburgh and Roe, 2011). Indeed, a large percentage of mediums have been found to be high functioning, socially accepted individuals within their communities (Krippner, 2007; Moreira-Almeida et al., 2007).

Most prior research on this phenomenon has focused on whether mediums can genuinely report accurate information under blinded conditions, and whether their personalities deviate in significant ways from population norms. But little is known about their physiological and electrocortical processes. Scientists have long proposed and used electroencephalography to study mediums in trance (deeply dissociated) states (Prince, 1968; Mesulan, 1981; Hughes and Melville, 1990; Oohashi et al., 2002; Hageman et al., 2010), but to our knowledge mental mediums who do not experience trance states have not been studied using these techniques. The present study investigated electrocortical activity in six professional mental mediums to explore two research questions: first, correlations between the accuracy of mediums' statements and their brain electrical activity were examined; and second, differences in mediums' brain activity were studied when they intentionally evoked four subjective states: perception, recollection, fabrication, and communication (as described below).

## Materials and methods

### Participants

Six individuals were randomly selected from a pool of 19 Windbridge Certified Research Mediums. These individuals were screened by the Windbridge Institute regarding their ability to report accurate and specific information about deceased individuals under blinded conditions. They also agreed to uphold a code of ethics and to volunteer their time to research (Beischel, 2007). Previous fMRI and other neuroimaging research has similarly used talented individuals to investigate mediumship (Mesulan, 1981; Hughes and Melville, 1990; Oohashi et al., 2002; Hageman et al., 2010; Peres et al., 2012).

Five females and one male participated in data collection. Their average age was 49.3 years and they had been participating in laboratory research for an average of 2.6 years. They each traveled from within the continental United States to the recording facility at the Institute of Noetic Sciences in Petaluma, California. They were not compensated for their time, but their travel expenses were reimbursed. Throughout this report, these six mediums are referred to by randomly assigned digits, e.g., Medium 1 or M1. The experimental protocols for both experiments were approved by the Institutional Review Board of the Institute of Noetic Sciences.

### Design

Sitters volunteered to participate in blinded reading sessions through a signup form on the windbridge.org research web page. Co-author MB screened sitters by email and phone prior to the experiment and asked them to provide the first names of deceased persons. A few days prior to data collection, MB provided the sitters with the approximate time at which the reading would take place and asked them to informally think about the deceased person at that time. At the start of each reading, the medium was given the first name of a deceased person and was asked to contact them. First names alone do not provide enough information to bias the medium's responses to specific questions about the deceased's personality, hobbies, profession, preferences, or cause of death (Beischel, 2007). The accuracy of the mediums' responses to these questions was later rated by the sitters who knew the deceased persons; these sitters were the only individuals with enough knowledge to effectively perform the accuracy rating task. The mediums and sitters never met each other and sitters were not present during the experiment.

All experimenters, mediums, and sitters were appropriately blinded to control for conventional explanations for the accuracy of the mediums' statements. This design eliminates the possibility of “cold reading” or fishing for information by the mediums. During the readings, an experimenter who did not know the deceased persons served as a proxy for the absent sitters and asked the questions to the mediums.

### Set-up

The mediums' electrocortical activity was recorded with a 32-channel EGI system (Net Amps 300 with 32 channel HydroCel™ Geodesic Sensor Net, Electrical Geodesics, Inc., Eugene, OR) at a sampling rate of 250 Hz. The default Cz electrode was used as a reference. Electrode impedance was maintained below 50 KOhm, which was the default impedance threshold on the EGI Net Amps 300 system. Electrode impedances were rechecked every 20 min and adjusted if necessary. Autonomic responses (i.e., galvanic skin conductance (GSR), respiration, heart rate, and blood flow) were recorded using a BIOPAC MP150 system (BIOPAC Systems Inc., Goleta, CA). Autonomic results will be reported in other publications.

During each data collection session, a medium and one experimenter (JB) were seated in a small (8' × 8') double steel-walled room. A microphone placed inside the room allowed a second experimenter (AD) in a nearby room to hear JB and the medium. AD manually marked events into the EEG records based on JB's verbal instructions, as described below. Mediums were instructed to keep their eyes closed for the duration of all of the recording sessions.

### Experiment 1

Each of the six mediums performed two blinded readings of two different deceased persons, for a total of 12 readings. Prior to each medium's two readings, experimenter JB was provided with the first names of the two deceased persons by MB, who had acquired the names from the associated sitters by phone during the screening process. MB had no contact with the mediums and was not present during the readings. Experimenter JB had no contact with the sitters and served as their proxy during the readings.

Mediums were given time to prepare for each reading; a few chose to meditate for about 1 min. Then JB provided him/her with the first name of a deceased person; the order of the two names read by each medium was randomized. All mediums claimed to be able to “connect” with the named deceased person in less than 1 min.

**Figure 1:**
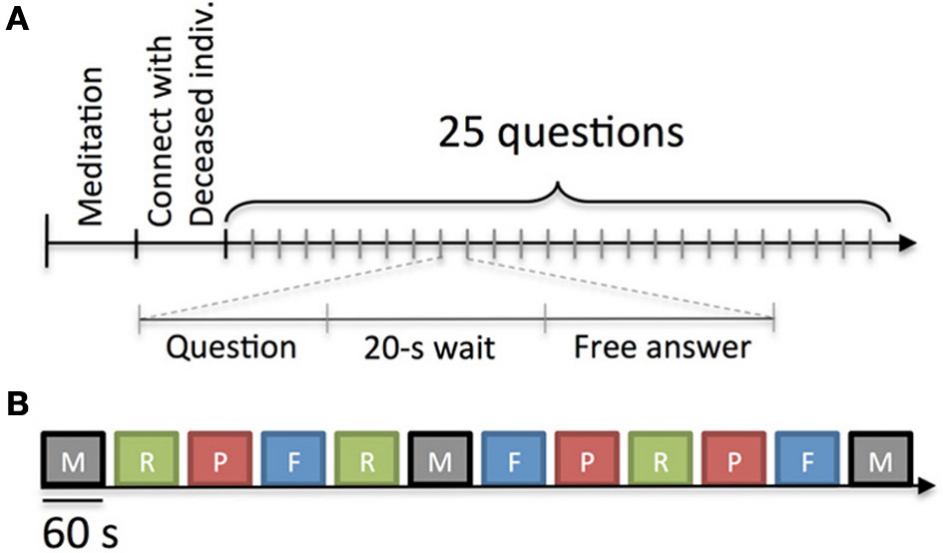
**Experimental protocols. (A)** Protocol for Experiment 1. After an optional meditation period of 1 min, the medium was given the first name of a deceased person with which to connect. All mediums claimed they connected with the deceased person within 1 min. Experimenter JB then asked the medium 25 questions. Each question was followed by 20 s of silence to provide for movement-free EEG data. **(B)** Protocol for Experiment 2. Mediums were asked to experience four different mental states: M, mediumship communication with a deceased person known to him/her; R, recollecting details about a living person known to him/her; P, perceiving biographical information about a deceased person read aloud by an experimenter; and F, fabricating a person and thinking about that imagined person. Each session lasted for 1 min and was followed by 15 s of rest. Each block of four sessions was repeated three times. The order of sessions was randomized within each block for each subject although a given state was never requested twice in a row. The figure depicts one possible order of experimental conditions.

During the reading, JB asked the medium 25 planned questions regarding aspects of the deceased's physical and personality characteristics, hobbies or interests, cause of death, preferences, experiences, and messages for the sitter (see Table A1). The order of the questions was randomized for each reading although questions belonging to similar categories were kept together and randomized within the category. After each question was asked, the medium remained quiet for 20 s to allow for movement-free EEG data to be collected. Investigator AD manually marked the EEG record after each question posed by JB. This was deemed appropriate since data analysis did not require fine temporal precision of the onset and offset of the 20-s response periods. JB notified the medium when this 20-s period was complete, and then the medium verbally answered the question (Figure 1A). The questions asked by JB and the mediums' responses were audio recorded for subsequent transcription.

It should be noted that these questions exceeded the standard question set used in previous research (Beischel, 2007) in both number (25 in the present case vs. 4–6 in other studies) and specificity. A larger number of questions were required in this study to collect sufficient EEG data, so questions about physical and personality characteristics, interests, and causes of death were divided into more specific sub-questions (see questions 1–18 in Table A1). Questions about aspects of the deceased persons' experiences and preferences were also added (see questions 19–25 in Table A1). In addition, in previous research mediums were able to respond to the questions immediately and provide as little or as much free-form information as they chose.

After the EEG data collection, JB transcribed the medium's responses to the 25 questions for each of the two readings and formatted the information into two lists of numbered items in which all references to the deceased persons' names were removed. The two lists were then randomized, identified by two consecutive numbers (e.g., Reading 5 and Reading 6), and emailed to a research assistant blinded to which readings were associated with which sitters and the identity of the medium who performed them. The research assistant then emailed each of the two associated sitters the two lists based on contact information obtained from MB. Later, the same research assistant received the scored readings from the sitters by email. During this procedure, each blinded sitter scored two readings (a target reading intended for him/her and a decoy reading intended for the other sitter) without knowing which was which. The 12 readings contained an average of 95 items each (range: 43–171 items).

The sitters rated the accuracy of each item in each list using a standardized scoring scale (Beischel, 2007). Mediums sometimes gave multiple answers to individual questions. Each of those items was rated independently and the percent accuracy (total items scored as “hits” divided by the total number of items minus those the sitter was unable to score) was calculated for each question.

Two of the 12 sitters did not return their scores. One had another loss in his/her family after the study readings took place and did not complete the scoring. Another sitter failed to respond despite repeated requests. Therefore, only data from 10 readings were available for analysis.

### Data analysis for experiment 1

EEG data were preprocessed by performing high pass filtering at 1Hz using a non-linear IIR filter (transition bandwidth of 0.3 Hz and order of 6). Zero to five channels containing large amplitude artifacts were removed by visual inspection of the power spectrum by investigator AD. AD also removed artifactual data segments by visual inspection. These segments contained paroxysmal activity due to head movement, electrical artifacts, or large amplitude eye movements. Minor eye movement artifacts were not removed. The data were converted to an average reference montage and Independent Component Analysis (ICA, Extended Infomax version) was applied to remove eye and muscle artifacts (Delorme and Makeig, 2004). Artifactual ICA components were visually identified by AD on the basis of scalp topography, time course, and power spectrum. Between one and six components were removed for each subject. When both sessions of Experiment 1 were performed without the medium removing the electrode cap, we used a single ICA decomposition for both sessions. If the medium removed the electrode cap between the two sessions to have lunch, we performed two separate ICA decompositions and rejection of ICA components.

After pre-processing the data, experimenter AD segmented data around each question using 20-s epochs: from 3 s before the end of the question to 17 s after the question. Any segment that had artifactual data as defined in the previous step was removed. Experimenter AD was blind to the accuracy ratings during the data-preprocessing phase.

The total number of data segments for the six mediums ranged from 24 to 42 (M1:29; M2:45; M3:24; M4:24; M5:35; M6:42). For some mediums (M3 and M4), scoring data from only one reading was available (as noted in the previous section). Data segments were classified into two categories: segments for which the accuracy of the response was high (i.e., rated above or equal to 50%) and segments for which the accuracy of the response was low (i.e., rated below 50%). High accuracy groups contained from 6 to 24 segments (M1:17; M2:22; M3:6; M4:15; M5:24; M6: 24) and low accuracy segments contained from 11 to 23 segments (M1:12; M2:23; M3:18; M4:9; M5:11; M6: 18).

The power spectrum was then computed for each data segment using a modified Welch method (Welch, 1967). A window of 1 s was used with 0% window overlap. The Welch method was modified to only consider every other window; that is, half of the windows were ignored to minimize autocorrelation between contiguous windows. It was found that for most of the mediums, there was no significant autocorrelation between neighboring selected windows in any of the frequency bands of interest. The absence of autocorrelation is important when performing statistical analyses which assume independence of samples. It may be noted that statistical methods and corrections for multiple comparisons do not always correct for autocorrelated EEG data, but given the controversial nature of the phenomenon under investigation, we chose to employ a more conservative approach. Weak autocorrelations between spectral estimates in neighboring windows remained for some mediums, but not for medium M1 whose data demonstrated the most significant effects.

Spectral power was then compared across all data channels between the two accuracy levels in four different frequency bands (theta 3–7 Hz; alpha 8–12 Hz; beta and low gamma 18–45 Hz; high gamma 75–110 Hz). These frequency bands were used because of their ubiquity in EEG research. We used a Monte-Carlo permutation method to compute statistics and the cluster method to correct for multiple comparisons across data channels (Maris et al., 2007). It should be noted that this method, which takes into account the structure of the data, such as the position of scalp electrodes, is the only valid method for correcting for multiple comparisons in EEG data since it accurately controls for type I errors. Finally, a conservative two-tailed threshold was set for significance at *p* = 0.01.

### Experiment 2

In Experiment 2, the mediums were asked to experience four different mental states: (1) Recollection (think about a living person known to the medium), (2) Perception (listen while an experimenter describes details about a person unknown to the medium), (3) Fabrication (think about a person imagined by the medium), and (4) Communication (interact mentally with a deceased person known to the medium). Each state was experienced for 1 min and mental states were experienced three times each. For the Perception condition, experimenter JB read details from biographies of deceased individuals collected online by a research assistant. The biographies included information similar in content to that requested from the mediums in Experiment 1. None of the mediums reported recognizing the persons depicted in the biographies. For the Communication and Recollection conditions, the medium chose deceased and living, respectively, friends or relatives prior to the beginning of the experiment.

The tasks in Experiment 2 were organized in 12 randomized and counter-balanced sessions of 1 min each (blocks of four conditions repeated three times) as shown in Figure 1B. For Recollection, Fabrication, and Communication conditions experienced the second and third times, the medium was asked to think about or connect with the same person as the one chosen for the first block. For subsequent blocks of the Perception condition, the experimenter continued reading from the biographies in 1-min segments. Between blocks, a 15-s break was taken so the mediums could transition more easily between mental states. Finally, a condition was never experienced twice in a row.

At the start of each 1-min block, JB instructed the mediums which mental state they should experience; she also notified them when to relax during breaks between conditions. JB also tracked the time for each condition and break between conditions using a silent stopwatch. As in Experiment 1, AD marked the EEG record according to JB's instructions.

### Data analysis for experiment 2

As in Experiment 1, the data were first preprocessed by performing high pass filtering at 1Hz using a non-linear IIR filter (transition bandwidth of 0.3 Hz and order of 6). Zero to five channels containing large amplitude artifacts were removed by visual inspection of the power spectrum by experimenter AD.

Fifty-second data segments were then extracted from 3 s before the end of the instructions to produce a specific mental state to 53 s after the instructions were complete. Since an experimenter manually entered the events, up to 1 s of inaccuracy was expected in terms of event latency. Extracting 50-s instead of 60-s data segments ensured that the medium was experiencing a specific mental state. Electrodes with artifactual data were removed by visual inspection by AD; artifactual data segments were also removed manually by AD from each 50-s epoch. The remaining data were then converted to average reference montage, Infomax ICA was performed, and artifactual components in each subject were removed; ICA components were selected by visual inspection by AD as in Experiment 1.

For each medium, data were compared across the four mental states in the four frequency bands studied in Experiment 1 (theta 3–7 Hz; alpha 8–12 Hz; beta and low gamma 18–45 Hz; high gamma 75–110 Hz). As in that experiment, data were divided into 1-s segments each separated by 2 s; we ignored every other second to reduce autocorrelation between neighboring spectral estimates.

For each frequency band, prior to studying differences between mental states, spectral power was detrended for all of the 1-s spectral estimates over the course of the full 15-min recording before comparing the four conditions. This minimized the effect of spectral power drifts in the signal that may artificially generate differences between conditions.

## Results

### Accuracy results (experiment 1)

Since the number of subjects (6) was too low to perform a group analysis, significance was computed separately for each medium. Scoring data from the readings performed by Mediums 3 and 4 were not included in these analyses because two sitter ratings could not be obtained (see Methods). Two sets of ratings for each reading were examined: a set of target accuracy ratings provided by the blinded sitter for whom the unidentified reading was intended and a set of decoy ratings provided by a second control sitter not associated with the reading.

For each of the four mediums for which both sitters returned their scores, the target and decoy ratings were compared. Two methods were used to compute significance, one based on non-parametric surrogate data analysis and one based on a Wilcoxon signed rank test. For the surrogate data analysis method, under the null hypothesis of no difference between the target and decoy reading ratings, we randomly shuffled corresponding scores between the target and decoy ratings. For the Wilcoxon test, the average accuracy on each question was compared between the decoy and the target readings. All statistics are one-tailed with the null hypothesis (H0) that the accuracy would be identical for the decoy and for the target reading, and the alternative hypothesis (H1) that the accuracy would be higher for the target than for the decoy reading.

Three of the four mediums showed a significant bias toward reporting information scored as more accurate by sitters scoring target readings than by sitters scoring decoy readings. Medium 2's results were significant only when considering Wilcoxon statistics, so that outcome might not be as robust as the data from Medium 1 and Medium 5 (see Table 1).

**Table 1:** **Percent accuracy and statistical significance for target vs. decoy ratings listed by medium**.

	**Accuracy**	**Accuracy**	**Accuracy**	**Surrogate**	**Wilcoxon**
	**target (%)**	**decoy (%)**	**difference (%)**	**(*p*)**	**(*p*)**
M1	55.5	41.3	+14.2	0.008	0.029
M2	44.9	41.8	+3.1	ns	0.004
M5	63.3	16.5	+46.8	<0.00005	0.00005
M6	45.1	39.5	+5.6	ns	ns

It should be noted that absolute accuracy values for target and decoy ratings are subjective and depend on the judgment of the sitters. Some sitters may give generally higher scores to all items and some may give generally lower scores to all items. Calculating the differences between decoy and target reading scores (“Accuracy difference” column), rather than examining absolute target accuracy scores, addresses this bias.

### Correlation between accuracy and electrocortical activity (experiment 1)

Exploratory analyses were performed in four frequency bands of interest: theta at 3–7Hz, alpha at 8–12 Hz, beta and high beta at 18–45 Hz, and gamma at 70–110 Hz. Two cases of significance within groups of electrodes were found: M1 in the theta frequency band and M3 in the alpha frequency band.

**Figure 2:**
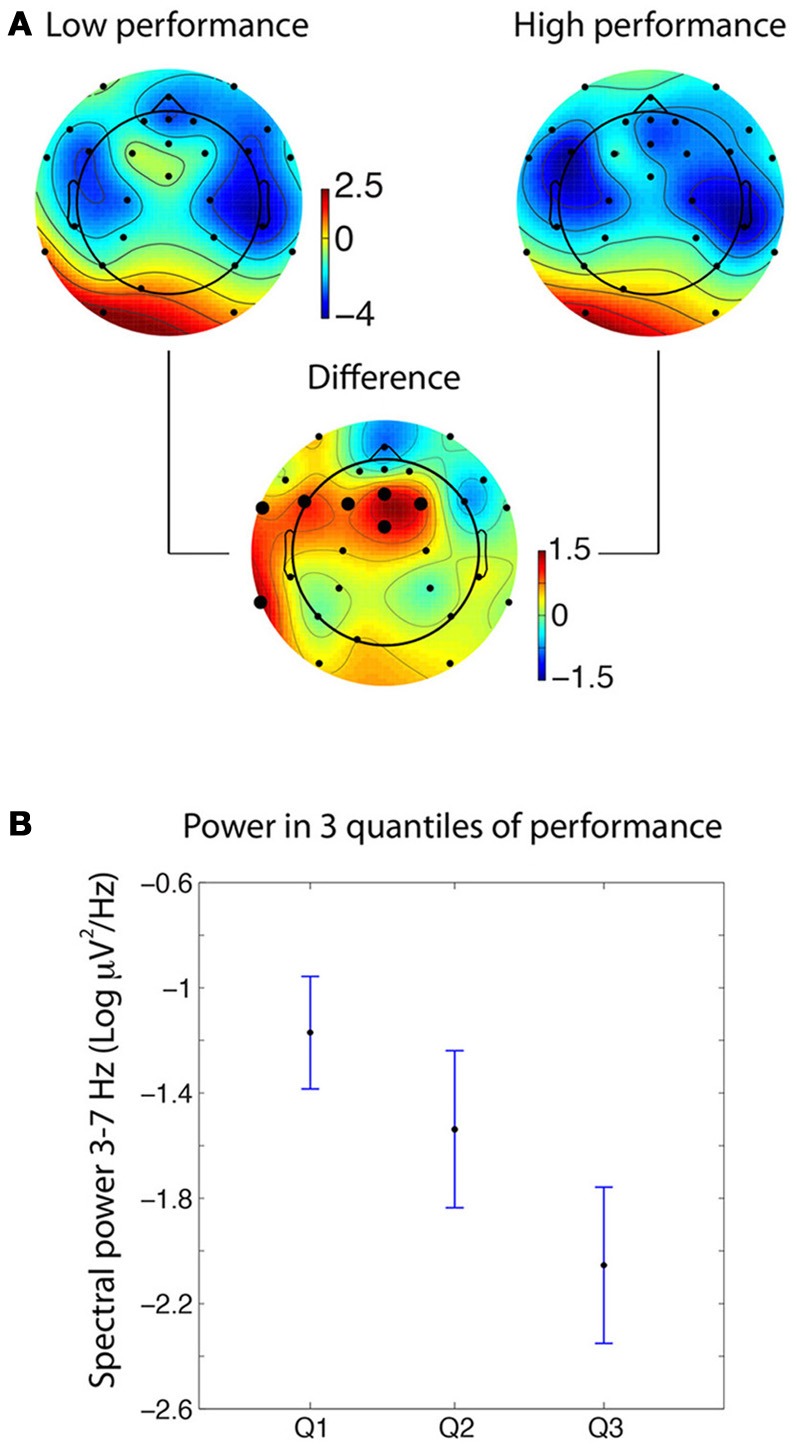
**Spectral differences in the theta frequency band for medium M1. (A)** Scalp topography of theta power during the mental experiences of information later rated as low (<50%) or high (≥50%) in accuracy [the unit being 10*log_10_(μV^2^)]. The large dots on the differential scalp topography indicate significance at *p* < 0.005. **(B)** When splitting the data into three quantiles of increasing accuracy (i.e., 0–34; 40–80; and 100% accuracy), theta power for the intermediate accuracy level fell in between the high and low accuracy levels. Error bars indicate 95% confidence intervals.

M1 demonstrated significant differences in frontal theta power between time periods rated as lower in accuracy (i.e., below 50%) compared to periods rated as higher in accuracy (i.e., equal to or above 50%). M3 showed similarly significant differences during low and high performance in the alpha frequency band. Results for M1 are shown in Figure 2.

For the significant electrodes, average power was computed in three quantiles containing about the same number of data segments (0–34% accuracy, 9 values; 40–80% accuracy, 9 values; 100% accuracy, 11 values). Figure 2B shows that the theta spectral power for the intermediate level of accuracy falls between the power for the low and the high accuracy segments. This reinforces the hypothesis that the effect we observe is linked to the medium's accuracy.

One explanation for this effect is that it is due to artifacts in the data. To test this hypothesis, the same analysis was run on the data artifacts instead of the cleaned data. Instead of considering the clean channel data with the ICA artifacts removed, only the artifactual data were considered, removing all non-artifactual ICA components from the raw data. Session 1 for Medium 1 had four artifactual ICA components and Session 2 for Medium 1 had three such artifacts. When processing artifactual data, no significant difference was observed between the low and high accuracy conditions. When looking at the three quantiles as in Figure 2B, the gradual decrease in power with theta accuracy was not observed.

Supplementary Figure 1 shows significant differences for M3 in the alpha 8–12 frequency band. M3 is one of the two mediums where only one reading was available instead of two, so this plot uses only about half of the data compared to the plot for M1. In contrast to M1 where theta power was negatively correlated with the medium accuracy, for M3 alpha power is positively correlated with accuracy. This result is consistent with the literature showing that alpha and theta power often tend to covary in opposite directions (Laufs et al., 2003; Braboszcz and Delorme, 2011). Since M3 only had one scored reading, there was not enough data to perform a quantile analysis as was done for Medium 1.

### Analysis of mental states (experiment 2)

**Figure 3:**
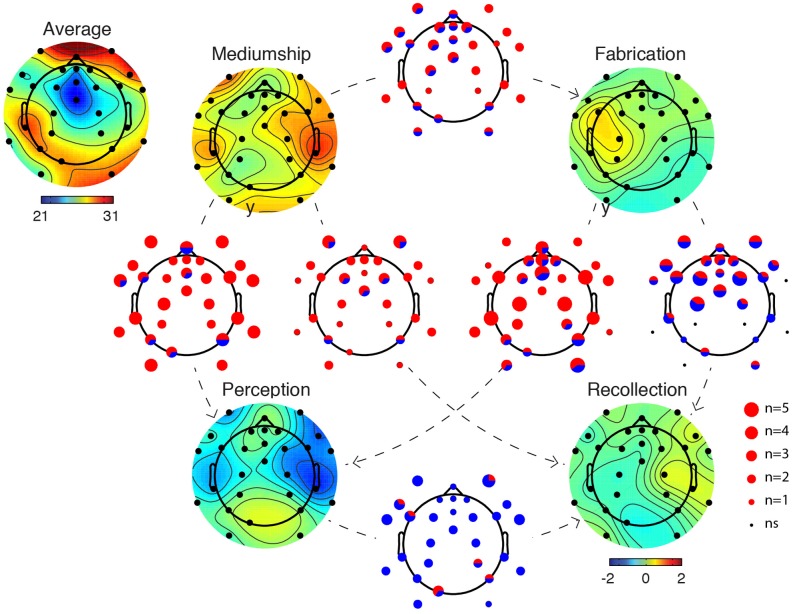
**Summary figures depicting differences between mental states in the high gamma frequency band (75–110 Hz)**. The scalp topography image in the left upper corner indicates the average gamma power for all mental states and all subjects [the unit being 10^*^log_10_(μV^2^)]. Scalp topographies for each mental state indicate deviation from the average scalp power (same units). In between each pair of mental states A and B, a plot indicates the number of significant participants for each electrode (*p* < 0.01 after cluster correction for multiple comparisons). The size of the dot represents the number of participants for which a given electrode is significant [from *n* = 0 (ns) to *n* = 5; significance in all six participants was never attained]. The pie chart within each dot represents the proportion of participants who demonstrated significant differences in one direction (red corresponds to A > B and blue corresponds to A < B). The direction of the comparison is indicated by a directional dashed line from A to B. For example, between the Mediumship Communication mental state and the Perception mental state, most electrodes are significant for three or four participants and the color (red) indicates that gamma power is of larger amplitude during the Communication mental state compared to the Perception mental state.

In Figure 3, EEG in the gamma frequency band for the four different mental states (Recollection, Fabrication, Perception, and Communication) for all six mediums is compared. We observed the largest number of significant electrode differences in the gamma frequency band (75–110 Hz). Figure 3 illustrates that the EEG is different in all conditions. Red dots on scalp maps indicate which electrodes are significant; Monte-Carlo surrogate statistics with cluster method were used to correct for multiple comparisons. The Mediumship Communication mental state differed more from the Perception mental state than from other mental states. For four of the mediums, larger amplitude high gamma power was observed during the Communication mental state as compared to the Perception mental state at nine electrode sites. The least consistent difference across mediums was the state of Fabrication compared to Recollection (Figure 3). For this pair of mental states, although most participants demonstrated a significant difference, about half of the mediums had higher gamma power in the Fabrication condition while the other half had higher power in the Recollection condition.

Gamma and high gamma activity in EEG is often questioned because muscle activity can contaminate this frequency band. Therefore, the same type of analysis was rerun but instead of processing the clean EEG data, only the ICA artifactual components were processed (Supplementary Figure 2). We found a pattern of gamma activity that was similar to Figure 3 in terms of the number of participants who showed significant differences and also in terms of the polarity of the difference. It was concluded that eye and muscle artifacts that were not successfully removed by ICA might dominate the difference observed in Figure 3. At this point, it cannot be concluded that the differences observed in Figure 3—albeit most likely genuine—originated from brain activity.

A number of significant effects in the beta and high beta frequency band (18–45 Hz) were also observed. These differences were similar to the differences observed in the gamma frequency band in terms of the polarity of the differences although fewer electrodes were significant. Table A2 summarizes the number of significant electrodes between each state for all mediums.

Differences were also found in the theta and alpha frequency bands although these differences were only present for three mediums (M1, M3, and M6) and only at specific locations on the scalp. In Supplementary Figure 3, details regarding which electrodes differed across the mental states in these frequency bands are illustrated. Few electrodes were significant in these frequency bands and the results were idiosyncratic across subjects.

## Discussion

A few prior electrophysiological (Solfvin et al., 1977; Mesulan, 1981; Hughes and Melville, 1990; Oohashi et al., 2002; Hageman et al., 2010) and neuroimaging (Peres et al., 2012) studies examined mediums or shamans in trance states. However, to the best of our knowledge, this experiment was the first EEG study of mental mediums who do not experience mediumship communication as a trance mental state. We observed decreased theta power in the frontal area during high accuracy segments of one medium's readings.

### Interpretation of the brain/accuracy correlation

Previous research with mediums has resulted in positive (e.g., Schwartz et al., 2002; Roy and Robertson, 2004; Beischel and Schwartz, 2007; Kelly and Arcangel, 2011) and negative (Schwartz et al., 2003; O'Keefe and Wiseman, 2005; Jensen and Cardeña, 2009) outcomes. Beischel and Schwartz (2007) performed an experiment similar in blinding to Experiment 1 with eight mediums and an experimenter serving as a proxy sitter in which blinded absent sitters scored target and decoy readings. Sitters provided significantly higher global scores for target readings than for decoy readings (*p* = 0.007) and sitters chose their target reading over a decoy reading 81% of the time when faced with a forced-choice task (*p* = 0.01). Kelly and Arcangel (2011) also performed an experiment employing a proxy sitter although they used a photograph of the deceased person instead of his first name and the proxy often knew personally or had information about the sitters and deceased individuals. In that study, each blinded sitter rated six transcripts. In the most successful experiment, sitters rated their reading in the top half (3 out of 6) in 30 cases out of 38 (*p* < 0.0001). Our significant accuracy results for mediums M1, M2, M5 and M6 are consistent with the results obtained in these studies.

As for the brain imaging data, our results from Medium 1 are also consistent with the previous literature. Activity in frontal areas demonstrated with fMRI has been associated with trance spiritual states (Jevning et al., 1996; Beauregard and Paquette, 2006). Decreased frontal midline theta rhythm might index decreased involvement of working memory neural circuits (Onton et al., 2005), which in turn might be consistent with a medium accessing a receptive mental state. We thus argue that these results could reflect genuine anomalous information transfer and would justify replication attempts with this medium.

### Correlation in the gamma frequency band

Findings in the EEG gamma frequency band have been associated with meditative trance states (Lutz et al., 2004; Cahn et al., 2010) and conscious thinking (Rieder et al., 2011). However, microsaccades seem to induce gamma in some EEG recordings (Yuval-Greenberg et al., 2008), which has raised doubts about all scalp EEG gamma findings. When observing potential gamma power chances, our approach in the past (Cahn et al., 2010) and in the present study was to perform the same analysis that was run on clean data on the artifacts. The fact that we found gamma power changes in the artifacts that resembled the findings in the clean data suggests that the results we observed are most likely not of brain origin. It could be, for example that the artifact extraction method failed to remove all eye movements and the origin of the gamma power changes we observed were movement artifacts.

The gamma differences between mental states seem to be similar in both data channels and artifacts. The artifacts we removed were most often eye-related activity and did not necessarily capture high frequency activity. We searched for common sources of noise that could contaminate brain activity in this frequency range. In particular we looked at galvanic skin response (GSR) and attempted to correlate GSR with gamma activity. The average GSR value was computed for each time window used to compute gamma power and the correlation was performed over all time windows irrespective of the mental state being experienced by the medium. Two participants showed significant correlation with GSR at five electrode sites at the *p* < 0.01 threshold after correction for multiple comparisons. However, given that this effect was not observed in all participants, it is unlikely that changes in GSR at the scalp level would be responsible for the changes in the gamma frequency band observed in most participants (Supplementary Figure 2).

### Study limitations

The design of this study, while necessary for the collection of clean EEG data in quantities large enough for analysis, was not conducive to the testing of hypotheses about mediums' ability to gain accurate information about deceased persons. First, these research mediums are not used to responding to multiple specific questions; in standard research protocols (Beischel, 2007), four to six questions are asked by the experimenter and each answer is then divided into items for rating purposes. In the current protocol, we asked a total of 25 questions. The periods of silence and stillness after questions were asked that were required to limit artifacts in the EEG recordings may also have been disruptive to the mediumship process. That is, after hearing a question, mediums were asked to experience information related to that questions silently for 20 s while EEG data were recorded. The items verbalized by the mediums at the end of those periods may not have reflected the full information they experienced during the collection of EEG data. In standard mediumship research readings, mediums are instructed to immediately “say what you see” and then provide an interpretation of the content if applicable. Although the speed with which each medium experiences and conveys information differs, generally speaking several items are experienced and spoken within any 20-s period. In the current study, information experienced during the 20-s pauses may have been forgotten or altered by the time it was verbalized. Thus, the correlation between cortical activity during the silent 20-s periods and the accuracy of the statements verbalized by the mediums after those periods is indirect at best.

## Conclusion

To conclude, we believe the results for Medium 1, correlating accuracy with electrocortical activity, qualify as a robust finding. The results regarding differences in gamma power bands between different mental states remains puzzling as the gamma difference we observed seems to arise, at least in part, from eye or muscular activity. The characterization of the exact nature of this difference in the gamma frequency band, and assessing whether any of this activity originates from the brain, calls for additional research. Taken together, the study's findings suggest that the experience of communicating with the deceased may be a distinct mental state that is not consistent with brain activity during ordinary thinking or imagination.

### Author contributions

Arnaud Delorme, Julie Beischel, Dean Radin, and Paul J. Mills designed the study. Julie Beischel recruited and trained the medium participants. Mark Boccuzzi recruited and trained the sitter participants. Julie Beischel, Leena Michel, Dean Radin, and Arnaud Delorme collected the EEG data and psychometric data. Julie Beischel, Mark Boccuzzi, and Arnaud Delorme analyzed the psychometric data. Julie Beischel and Arnaud Delorme analyzed the accuracy data. Arnaud Delorme analyzed the EEG data. Arnaud Delorme and Julie Beischel wrote the manuscript. Dean Radin and Paul J. Mills edited the manuscript.

### Conflict of interest statement

The authors declare that the research was conducted in the absence of any commercial or financial relationships that could be construed as a potential conflict of interest.

## Figures and Tables

**Table A2:**
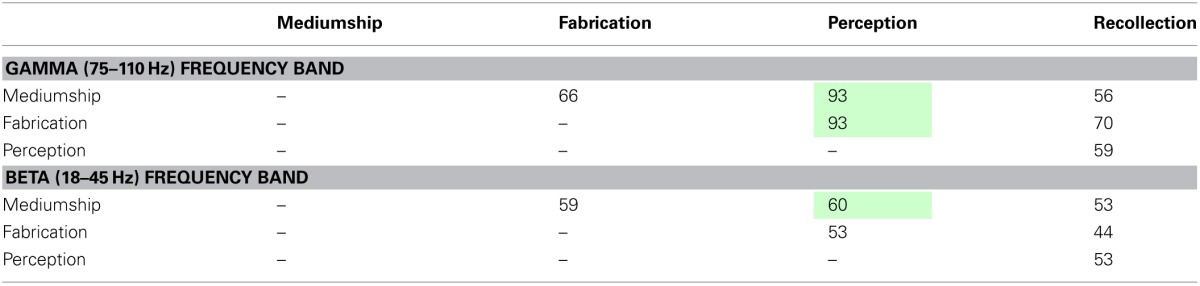
**Comparison of the cumulative number of significant electrodes across the six participants between mental states in the gamma and beta frequency bands**.

The maximum for each table is highlighted using a green background.

## Supplementary material

Figure S1 **Correlation between alpha power (8–10 Hz) and for the accuracy of statements reported by Medium 3**. Legend same as for Figure 2A.

Figure S2 **This figure is similar to Figure 3 but instead of processing the data, we processed artifacts as isolated by independent component analysis**.

Figure S3 **Grand average spectrum for three participants that showed significant differences between mental states [the unit being 10*log_**10**_(μV^**2**^)]**. The ANOVA method was used to compute significance. Electrodes in white indicate those deemed significant after correction for multiple comparison and across all mental conditions.

## Appendix

**Table A1:** **Questions asked of each medium during each reading**.

**Questions regarding the discarnate's appearance during his/her physical life:**
1. Describe the discarnate's hair. 2. Describe the discarnate's eyes. 3. Describe the discarnate's build. 4. Describe the discarnate's height. 5. About how old was the discarnate when s/he passed away? 6. Is there anything else you can tell me about the discarnate's physical appearance?
**Questions regarding the discarnate's personality:**
7. Was the discarnate more shy or more outgoing? 8. Was the discarnate more serious or more playful? 9. Was the discarnate more rational or more emotional? 10. Is there anything else you can tell me about the discarnate's personality?
**Questions regarding the discarnate's hobbies/activities/interests:**
11. Did s/he spend more of his/her free time indoors or outdoors? 12. Were most of his/her activities solitary or were most social? 13. Were most of his/her activities physical or were most not physical? 14. Is there anything else you can tell me about the discarnate's hobbies/activities/interests?
**Questions regarding the discarnate's cause of death:**
15. Did the death occur quickly or slowly? 16. Was the cause of death natural or unnatural? 17. What body parts were affected at the death? 18. Is there anything else you can tell me about the discarnate's cause of death?
19. **What were the discarnate's favorite foods?**
20. **What was the discarnate's occupation? What did s/he do for a living?**
21. **Provide dates or times of year important to the discarnate**.
22. **Provide names of people who were close or important to the discarnate**.
23. **Describe any animals that were close or important to the discarnate**.
24. **Does the discarnate have any messages specifically for the sitter?**
25. **Is there anything else you can tell me about the discarnate?**

Categories are listed in bolded text. For each reading, categories and sub-questions within each category (where applicable) were randomized, However, the final sub-question in a category (i.e., questions 6, 10, 14, and 18) and the final question overall (i.e., question 25) were asked last for each category and overall, respectively. Questions were worded to reflect the deceased person's gender (e.g., ‘What did she do for a living?’ or ‘Were most of his activities solitary or were most social?’). In this questionnaire, the deceased person is designated by the label “discarnate,” a common term used in mediumship research.
