# Supplementary figures and images for: Electrocortical activity associated with subjective communication with the deceased
Source: Front Psychol. 2013 Nov 20;4:834. doi: 10.3389/fpsyg.2013.00834 (PMC3834343; doi:10.3389/fpsyg.2013.00834)

Low performance

High performance

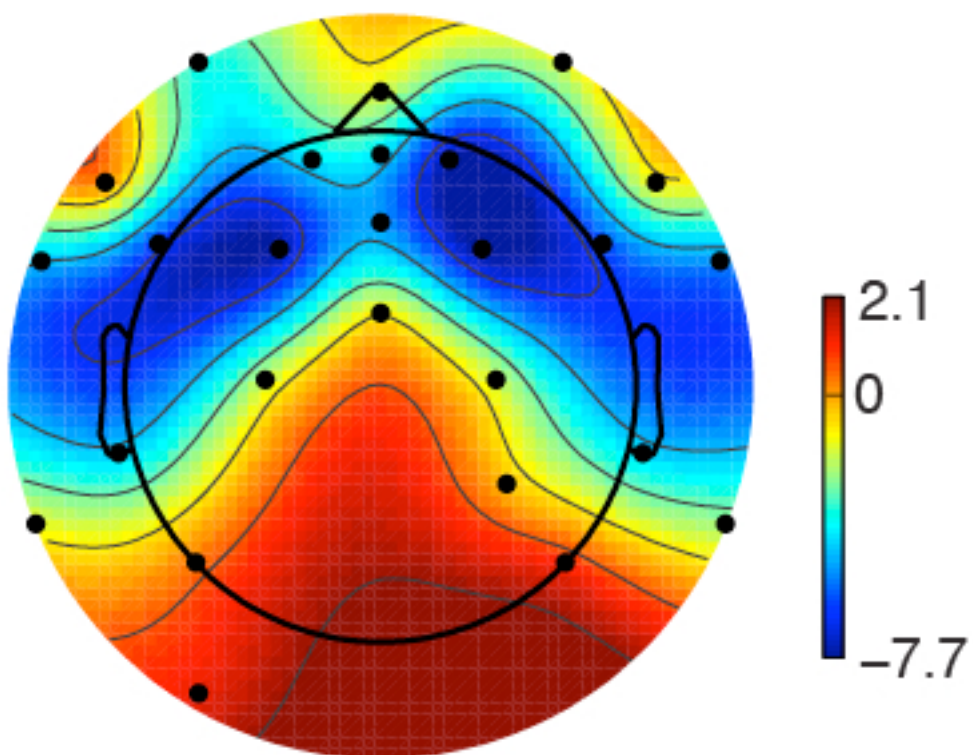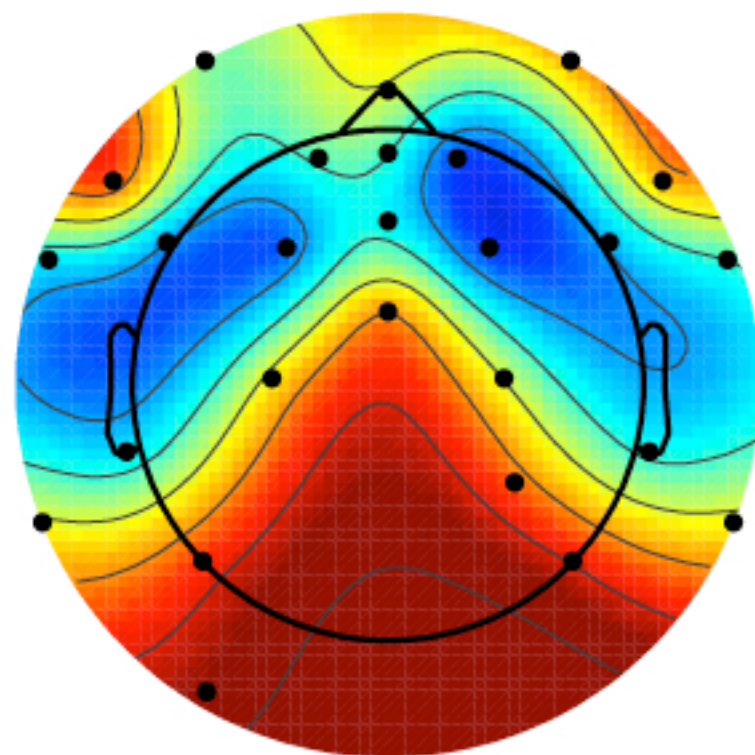

Difference

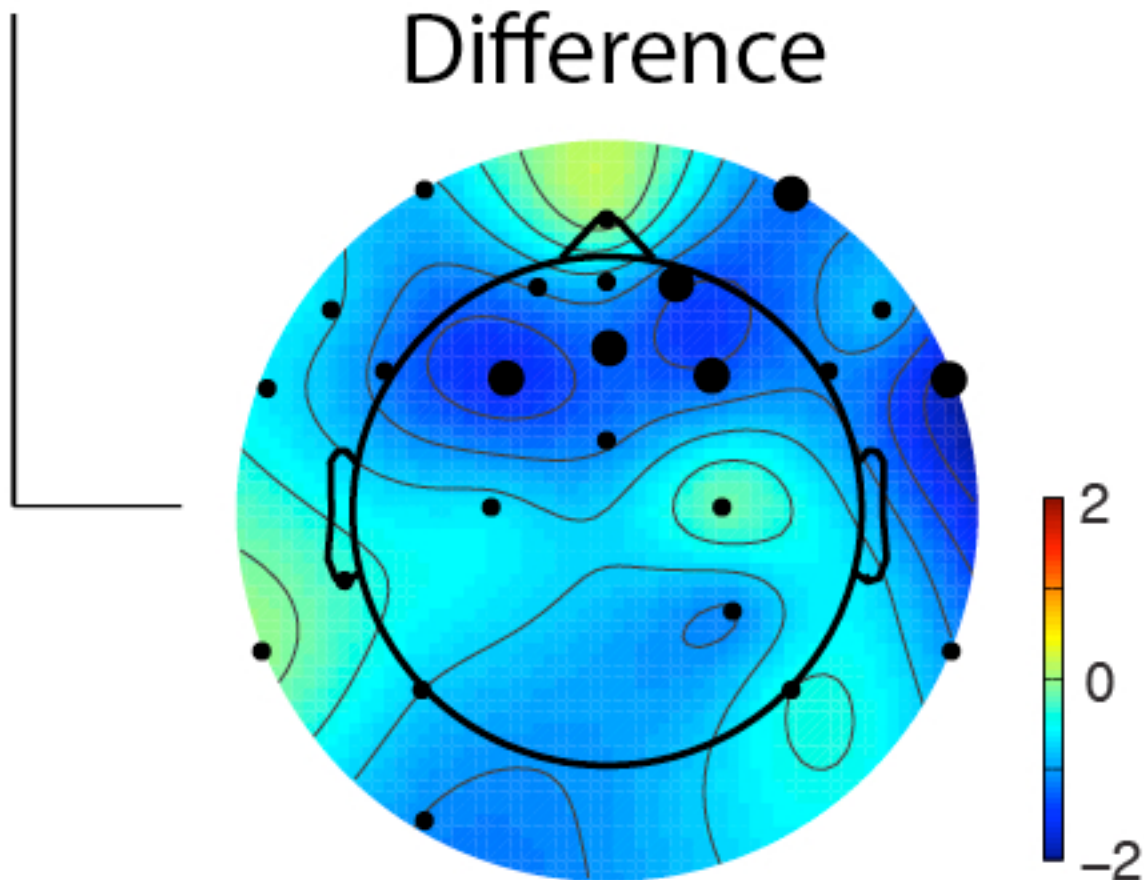

Supplement: Figure S1 — Correlation between alpha power (8–10 Hz) and for the accuracy of statements reported by Medium 3. Legend same as for Figure 2A. [file Presentation1.PDF]

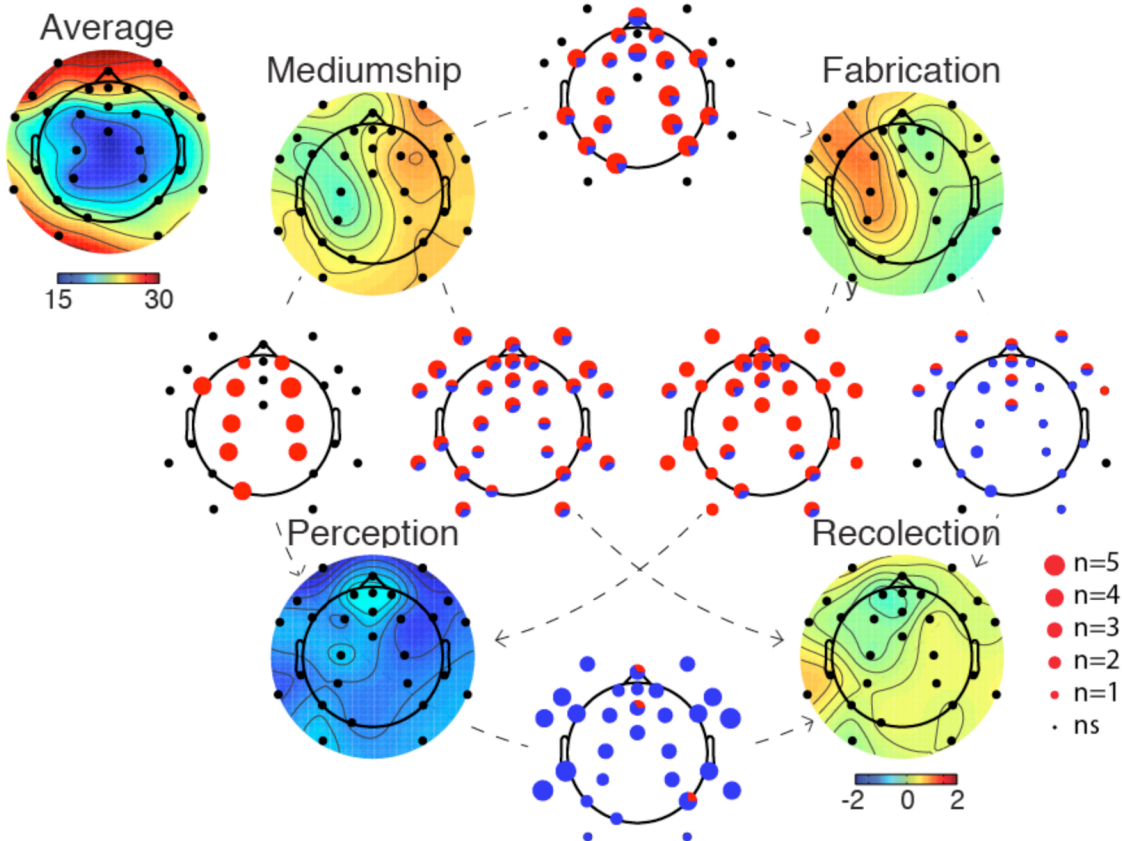

Supplement: Figure S2 — This figure is similar to Figure 3 but instead of processing the data, we processed artifacts as isolated by independent component analysis. [file Presentation2.PDF]

Theta 5-7Hz

Alpha 8-12Hz

M1

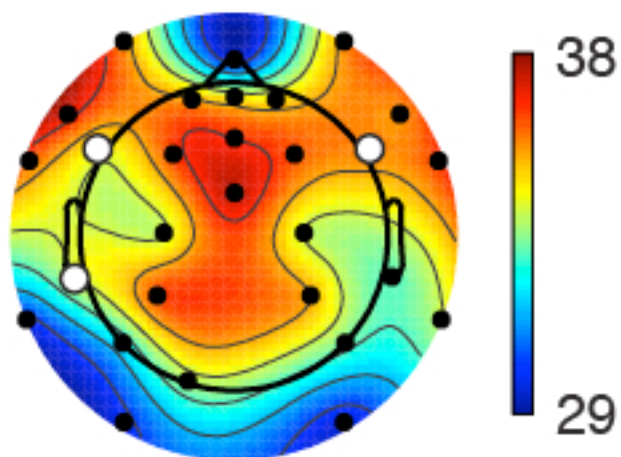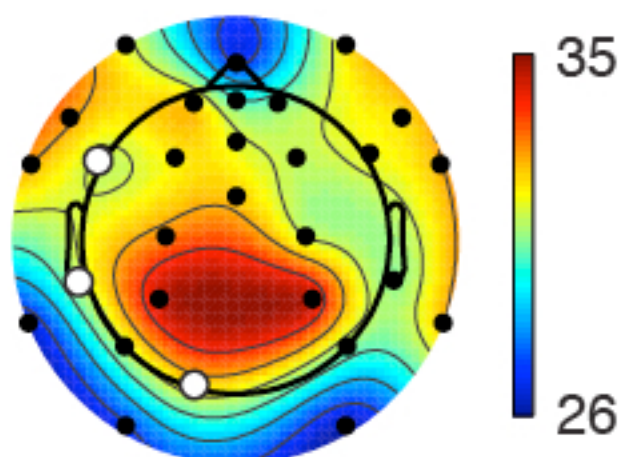

M4

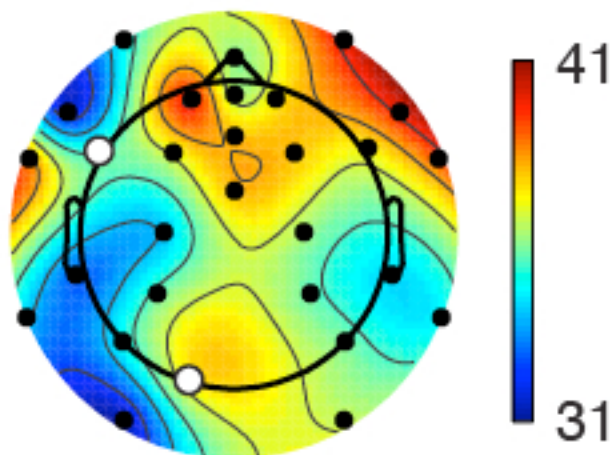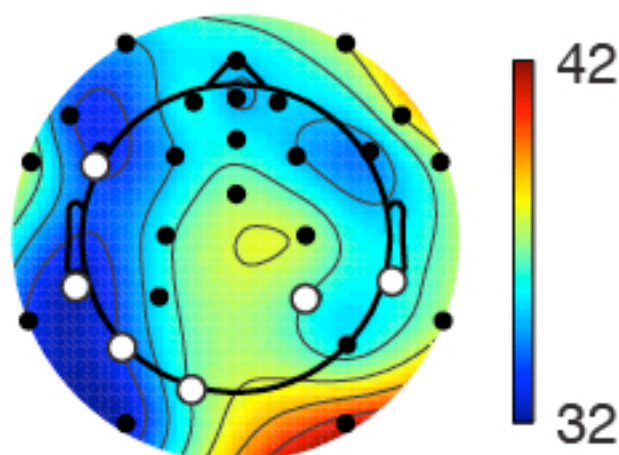

M6

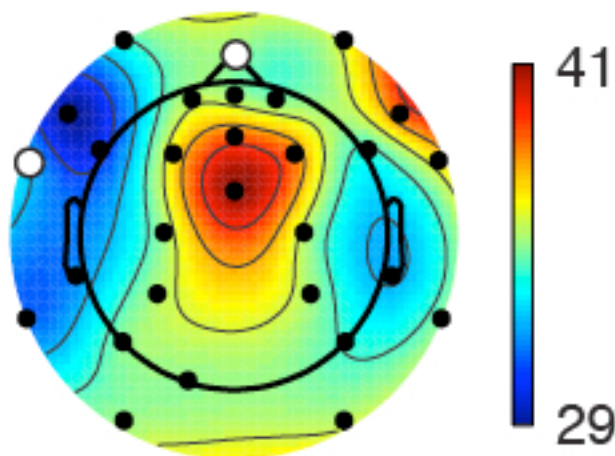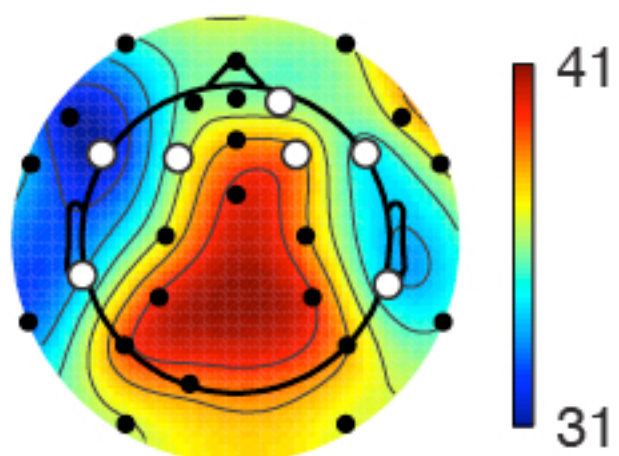

Supplement: Figure S3 — Grand average spectrum for three participants that showed significant differences between mental states [the unit being 10*log10(μV2)]. The ANOVA method was used to compute significance. Electrodes in white indicate those deemed significant after correction for multiple comparison and across all mental conditions. [file Presentation3.PDF]
